# Primary Pure Squamous Cell Carcinoma of the Gallbladder Locally Invading the Liver, Duodenum, and Stomach: A Case Report and Literature Review

**DOI:** 10.1155/2017/2534029

**Published:** 2017-01-29

**Authors:** Aldrin C. Alpuerto, Maximo E. Mora, R. Jonathan Robitsek, Sebastian D. Schubl

**Affiliations:** ^1^Northeast Ohio Medical University, Rootstown, OH 44272, USA; ^2^Department of Pathology, Jamaica Hospital Medical Center, Jamaica, NY 11418, USA; ^3^Department of Surgery, Jamaica Hospital Medical Center, Jamaica, NY 11418, USA; ^4^Department of Surgery, University of California Irvine Medical Center, Orange, CA 92868, USA

## Abstract

Primary pure squamous cell carcinoma (SCC) of the gallbladder is an exceptionally rare type of tumor that comprises only 1% of all gallbladder cancer. SCC of the gallbladder portends a worse prognosis than the more common adenocarcinoma variant because of its aggressive invasion to local structures and because it is often diagnosed at an advanced stage. Owing to its rarity, diagnosis and management can be challenging. Herein, we present the case of a 75-year-old female complaining of abdominal pain, nausea, and vomiting. Computed tomography and ultrasonography results of the abdomen were consistent with acute cholecystitis and cholelithiasis. Histologic evaluation of the resected mass revealed a malignant tumor with prominent keratinization, confirming the diagnosis of an invasive primary pure SCC of the gallbladder. Microscopic examination showed direct infiltration to the liver, duodenum, and stomach. This case report describes the hospital course of a patient with SCC of the gallbladder and suggests that gallbladder cancer should be considered as part of the differential diagnosis in elderly patients presenting with acute cholecystitis. In addition, this article will review existing literature to examine the utility of different diagnostic techniques and treatment modalities available in the management of gallbladder cancer.

## 1. Introduction

Primary pure SCC of the gallbladder is a locally invasive cancer characterized by prominent keratinization, in the form of keratin pearls, without evidence of malignant glandular transformation [1]. This subtype of gallbladder cancer (GBC) is theorized to originate either from squamous metaplasia of an existing adenocarcinoma or from a metaplasia-dysplasia-carcinoma sequence [1–7].

Abdominal ultrasonography (U/S) is typically the first diagnostic procedure performed for suspected biliary disease [8]. However, GBC can only be confirmed by histology [1]. The prognosis of gallbladder cancer is dismal. Due to the paucity of cases reported, clear guidelines on therapeutic management are not well defined. Surgical intervention offers some possibility of long-term survival [9]. The efficacy of radiotherapy and chemotherapy is debatable, although it may have some role in palliative management [10, 11].

## 2. Case Report

**Figure 1:**
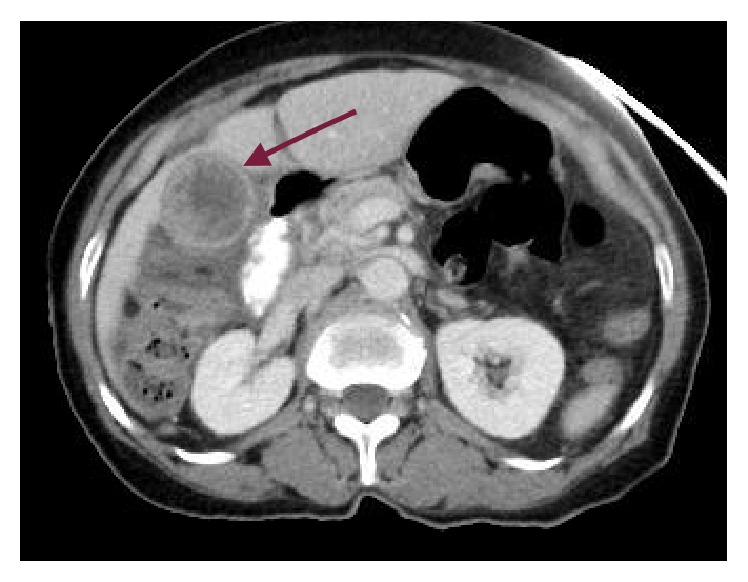
Abdominal CT showing irregular tissue mass in the gallbladder fundus (arrow).

**Figure 2:**
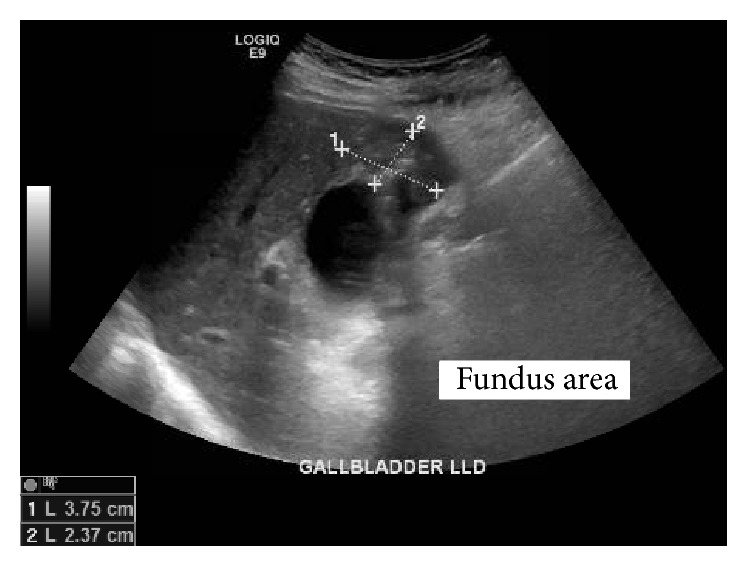
Right upper quadrant ultrasound showing 3.8 × 2.4 × 3.5 heterogeneous mass with small calcification emanating from the gallbladder fundus.

A 75-year-old Haitian female presented in September 2015 complaining of abdominal pain, which worsened with food intake. She reported that her pain was associated with nausea and vomiting. Abdominal examination was remarkable for tenderness to palpation in the right upper quadrant. Further work-up revealed leukocytosis (15,000 WBC/*µ*L) with neutrophilic predominance. The liver enzyme test results were within normal limits. Computed tomography (CT) demonstrated gallbladder wall thickening and pericholecystic fluid suggestive of acute cholecystitis (Figure 1). Transabdominal U/S showed a 3.8 × 2.4 × 3.5 cm heterogeneous lesion emanating from the gallbladder fundus, suspicious for malignancy (Figure 2). A percutaneous cholecystostomy catheter was inserted and 60 mL of brown-colored aspirate was obtained. Cytologic study of the sample revealed rare ductal cells, few atypical cells, and anucleated squamous cells in a proteinaceous background. After several days, the patient was discharged home.

**Figure 3:**
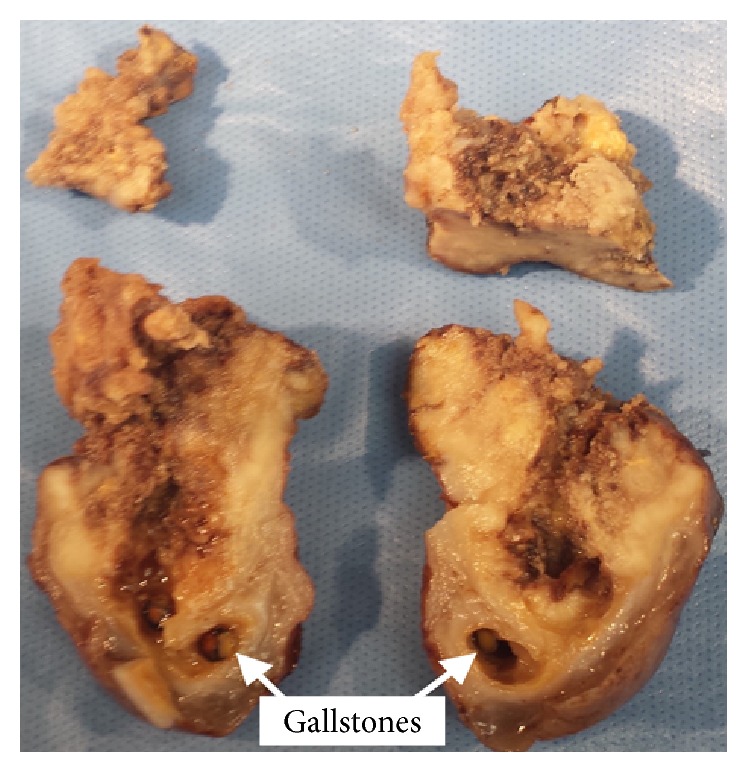
Gross examination reveals diffuse gallbladder wall thickening from the tumor mass encompassing the entire gallbladder lumen.

**Figure 4:**
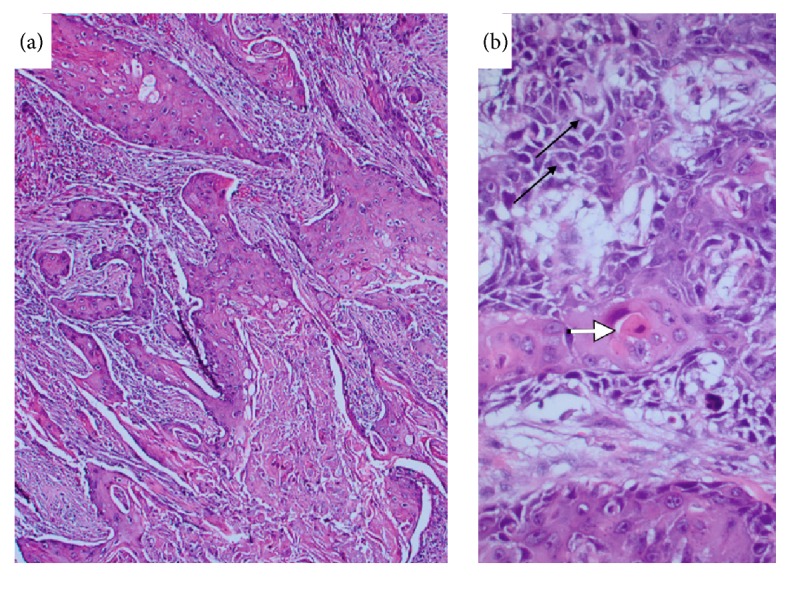
Microscopic examination of gallbladder shows infiltrating carcinoma (a). Tumor is composed of squamous cells with individual cell keratinization (white arrow) and intercellular bridges (black arrows) (b).

**Figure 5:**
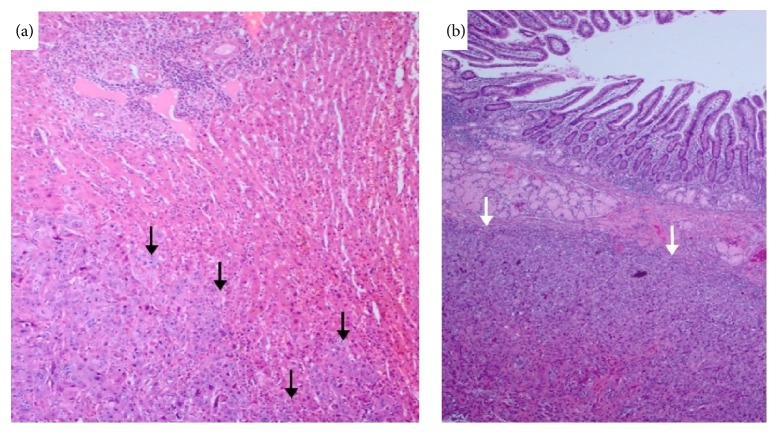
Local invasion of the tumor in liver parenchyma (black arrows) (a) and duodenum (white arrows) (b).

In December 2015, the patient returned to the hospital after noticing serosanguinous fluid draining from the cholecystostomy tube. A cholecystogram revealed cystic duct obstruction. In January 2016, the patient underwent an elective laparoscopic cholecystectomy, which was converted to an open cholecystectomy. Intraoperatively, the gallbladder was noted to be chronically inflamed. A choleduodenal fistula involving the gallbladder fundus and the first part of the duodenum was identified. The gallbladder and distal stomach were resected and sent to pathology. On gross pathology, the fundal mass measured 5.5 cm at its greatest dimension. The tumor appeared to have diffuse growth encompassing the entire gallbladder lumen (Figure 3). The pathology report diagnosed a stage T4NxMx, invasive, moderately differentiated squamous cell carcinoma. Prominent keratin pearls and intercellular bridges were evident in histology, which are characteristic of squamous cell differentiation (Figure 4). The serosal surface was positive for malignancy. Direct infiltration of the hepatic parenchyma and duodenum was noted (Figure 5). The distal stomach specimen was positive for invasive squamous cell carcinoma involving the serosa and muscularis propria of the duodenum. The proximal and distal margins of the resection were positive for malignancy. The remaining gastric mucosa showed chronic active gastritis. The cystic duct was negative for malignancy with reactive glandular atypia. The patient did well postoperatively and denied any complaints during her 1-month follow-up.

## 3. Discussion

### 3.1. Epidemiology

Gallbladder cancer is rare. In a population-based study conducted by the Centers for Disease Control and Prevention (CDC), the incidence rate of primary GBC in the United States was 1.13 per 100,000 persons per year from 2007 to 2011. The incidence of gallbladder cancer is higher in women than in men and higher in American Indians than in non-Hispanic Caucasians [12]. The incidence is greater in South American countries, particularly Chile, and in Asian countries, especially India and Japan. Delhi, India, has the highest gallbladder cancer incidence rates for women (21.5 per 100,000) [13]. Few cases have been reported from the United States.

Pure primary squamous cell carcinoma is an exceptionally rare subtype of GBC, making up only 1% of all malignant gallbladder neoplasm according to a cohort study on 606 invasive gallbladder carcinomas [1]. However, a wide range of incidence rates has been reported. Some of these data were confounded by the inclusion of mixed adenosquamous carcinoma, leading to a discrepancy in incidence rate [14]. Roa et al. clearly defined pure SCC of the gallbladder as invasive carcinoma composed solely of squamous differentiation without any invasive glandular component. With this definition, an incidence rate of 1% of all GBC was determined [1].

### 3.2. Prognosis

Roa et al. evaluated the clinicopathological characteristics of mixed adenosquamous carcinoma/SCC variant compared to adenocarcinoma of the gallbladder. In that study, the mean survival time of the adenosquamous carcinoma/SCC variant was 23 months (range, 1–112.5 months; median, 4 months). In comparison, adenocarcinoma of the gallbladder was 50 months (range, 1–160 months; median, 12 months) [1].

### 3.3. Etiology

The etiology of the SCC subtype of gallbladder cancer is unknown. Several theories have been proposed. One theory is that existing adenocarcinoma undergoes squamous metaplasia [1–4]. In this theory, the squamous cell component of mixed adenosquamous gallbladder carcinoma undergoes overgrowth and eventually replaces all of the adenocarcinoma elements, thus forming a pure SCC [4, 5]. Another theory describes a progression of metaplasia-dysplasia-carcinoma sequence. This suggests that chronic irritation from gallstones may trigger differentiation of gallbladder glandular cells into squamous cells. These squamous metaplastic cells would then undergo malignant transformation [1–7]. In agreement with the latter theory, Roa et al. reported that approximately 12% of gallbladder cancer has squamous metaplastic cells evident in adjacent mucosa [1].

### 3.4. Diagnosis

A definitive diagnosis of GBC is confirmed by histopathological evaluation of the resected mass. Consequently, most cases are not diagnosed preoperatively [1]. Typically, a gallbladder mass is found incidentally in patients presenting with nonspecific signs and symptoms that mimic acute cholecystitis [1].

Abdominal U/S is the initial diagnostic procedure of choice for biliary diseases. However, radiologic studies may be limited in a setting where gallbladder cancer has overlapping symptoms with cholecystitis. Liang et al. reported a retrospective study of 26 patients with confirmed GBC, who initially had presumptive diagnosis of acute cholecystitis based on U/S. Of the 26 patients, 15 (57.6%) exhibited diffuse gallbladder wall thickening and 11 (42.3%) exhibited focal thickening or an intraluminal mass [8]. In our case as well, radiological study was consistent with acute cholecystitis. Thus, in elderly patients with radiologic findings of diffuse gallbladder wall thickening and intraluminal masses, a diagnosis of gallbladder cancer should be considered.

Cytology of bile samples often results in variable findings, and sensitivities are limited because of sample degeneration [15]. However, P. Gupta and R. K. Gupta reported 9 cases of SCC of the gallbladder diagnosed by use of U/S-guided aspiration cytology out of 322 cases with gallbladder mass lesions (2.8%) [16]. In the present case report, the cytology report from the cholecystostomy drain noted atypical cells and anucleated squamous cells. This may suggest that evidence of squamous cells in aspirated biliary fluid, which should be composed entirely of glandular cells, is suspicious for squamous cell carcinoma. Thus, cytology studies of biliary fluid should be further investigated as a potential means of making a preoperative diagnosis of SCC of the gallbladder.

U/S-guided FNA biopsy has shown promise as a minimally invasive tool to diagnose GBC, preoperatively. Zargar et al. performed U/S-guided FNA biopsy on 88 patients with gallbladder masses. GBC was confirmed in 69 of the 78 cases (88.5%) suspected to be malignant. The remaining 10 cases (100%) were accurately diagnosed as benign [17]. Moreover, one case report described the use of U/S-guided FNA with Rapid On-Site Evaluation (ROSE), which yielded numerous malignant cells with dense waxy cytoplasm, pleomorphic nuclei, and high nucleus-to-cytoplasm ratio. This suggests the diagnosis of GBC. In that case, the early detection allowed surgical intervention without delay. If suspicion for GBC is high, Chambers et al. suggest that FNA with ROSE may be part of the first-line test to confirm the diagnosis [18].

### 3.5. Treatment

Therapeutic strategy for gallbladder cancers has not been established. Surgical intervention offers the only possibility for curative therapy. Kobayashi et al. reported 2 patients with advanced SCC of the gallbladder, who survived 9 and 10 years without recurrence after radical resection [9]. However, reports of long-term survival after surgical resection are anecdotal at best [19]. In the early stages, cholecystectomy may be adequate. Late stages with local invasion may require radical dissection [2, 10]. Metastatic disease and peritoneal dissemination are poor prognostic factors and such cases are not amenable to surgical resection. Unfortunately, most patients have unresectable GBC at presentation. Only 10% to 30% of patients are candidates for surgery [11]. In our case, a distal gastrectomy with Billroth II procedure, in addition to the cholecystectomy, was justified because of the gastric and duodenal involvement.

Adjuvant chemotherapy and radiotherapy yielded inconsistent outcomes but may have some use in palliation of advanced cancer. Mallick et al. reported the use of both external beam radiotherapy (45 Gy) and chemotherapy (5-fluorouracil with leucovorin) in three patients. These treatments were given 4 to 6 weeks after their surgery. One patient developed lung and spleen metastases, another was lost to follow-up, and the progress of the third patient was not detailed in the case report [10]. Further literature review revealed no other precedent for use of chemotherapeutic agents. More promisingly, Hou et al. reported one case in which a patient had complete tumor remission 2 months after treatment with radiotherapy alone. Unfortunately, the patient ultimately died of liver metastasis 15 months after the radiotherapy [11].

## 4. Conclusion

Pure primary SCC of the gallbladder is a rare type of gallbladder cancer. Abdominal U/S and CT are the initial diagnostic tests used for biliary disease. GBC should be considered in elderly patients with radiologic evidence of diffuse gallbladder wall thickening and intraluminal masses. Cytology of biliary fluids and U/S-guided FNA are potentially safe tools to diagnose GBC. However, this requires further investigation. Although no therapeutic strategy has been defined, surgery offers the only possibility for a cure. The benefits of chemotherapy and radiotherapy are unclear and should be explored.

## References

[1] Roa J. C., Tapia O., Cakir A. (2011). Squamous cell and adenosquamous carcinomas of the gallbladder: clinicopathological analysis of 34 cases identified in 606 carcinomas. *Modern Pathology*.

[2] Khan N., Afroz N., Haider N., Khan M. A. (2012). A case of pure endophytic squamous cell carcinoma of the gallbladder: a rare entity with aggressive behaviour. *Turkish Journal of Pathology*.

[3] Roppongi T., Takeyoshi I., Ohwada S. (2000). Minute squamous cell carcinoma of the gallbladder: a case report. *Japanese Journal of Clinical Oncology*.

[4] Rao S., Arya A., Aggarwal S., Gupta K., Arora R., Dhawan I. (2007). Pure squamous cell carcinoma of the gall bladder. *Indian Journal of Pathology and Microbiology*.

[5] Chakrabarti I., Giri A., Ghosh N. (2014). Cytohistopathological correlation of a case of squamous cell carcinoma of gallbladder with lymph node metastasis. *Turk Patoloji Dergisi*.

[6] Khaira H. S., Awad R. W., Thompson A. K. (1995). Squamous cell carcinoma of the gallbladder presenting with a biliary-colic fistula. *European Journal of Surgical Oncology*.

[7] Al-Hady S. M., Al-Saed A. (1997). Primary keratinizing squamous cell carcinoma: an exceptional tumor of the gallbladder. *Saudi Journal of Gastroenterology*.

[8] Liang J.-L., Chen M.-C., Huang H.-Y. (2009). Gallbladder carcinoma manifesting as acute cholecystitis: clinical and computed tomographic features. *Surgery*.

[9] Kobayashi A., Miyagawa S., Miwa S. (2007). Radical surgery for advanced squamous cell carcinoma of the gallbladder: a report of three cases, including a 10-year survivor. *Hepato-Gastroenterology*.

[10] Mallick S., Benson R., Julka P. K., Rath G. K. (2014). Adjuvant chemoradiotherapy for squamous cell carcinoma of gallbladder. *Journal of Gastrointestinal Cancer*.

[11] Hou J., Zeng Z., Sun J., Ji Y. (2010). Conformal radiotherapy for squamous cell carcinoma of gallbladder: a case report. *Case Reports in Medicine*.

[12] Henley S. J., Weir H. K., Jim M. A., Watson M., Richardson L. C. (2015). Gallbladder cancer incidence and mortality, United States 1999-2011. *Cancer Epidemiology Biomarkers and Prevention*.

[13] Randi G., Franceschi S., La Vecchia C. (2006). Gallbladder cancer worldwide: geographical distribution and risk factors. *International Journal of Cancer*.

[14] Hosseinzadeh M., Shokripur M., Salahi H. (2012). Primary pure squamous cell carcinoma of gallbladder presenting as acute cholecystitis. *Iranian Journal of Medical Sciences*.

[15] Lam C. M., Yuen A. W., Wai A. C. (2005). Gallbladder cancer presenting with acute cholecystitis: A Population-Based Study. *Surgical Endoscopy and Other Interventional Techniques*.

[16] Gupta P., Gupta R. K. (2012). Preoperative diagnosis of squamous cell carcinoma of the gallbladder by ultrasound-guided aspiration cytology: clinical and cytological findings of nine cases. *Journal of Gastrointestinal Cancer*.

[17] Zargar S. A., Khuroo M. S., Mahajan R., Jan G. M., Shah P. (1991). US-guided fine-needle aspiration biopsy of gallbladder masses. *Radiology*.

[18] Chambers M. R., Hasan M. K., Hébert-Magee S. (2016). Pearls before bile: primary squamous cell carcinoma of the gallbladder diagnosed on-site by endoscopic ultrasound-guided fine-needle aspiration. *Digestive Endoscopy*.

[19] Soyama A., Tajima Y., Kuroki T. (2011). Radical surgery for advanced pure squamous cell carcinoma of the gallbladder: report of a case. *Hepato-Gastroenterology*.

